# A comparative study of postadrenalectomy hyperuricemia and renal impairment in patients with unilateral primary aldosteronism: does histopathology subtype matter?

**DOI:** 10.1186/s12882-024-03750-4

**Published:** 2024-10-13

**Authors:** Chu-Wen Fang, Hui-Lung Hsieh, Shuo-Meng Wang, Kuo-How Huang, Kang-Yung Peng, Yen-Hung Lin, Vin-Cent Wu, Jeff S. Chueh

**Affiliations:** 1 Division of Urology, Department of Surgery, Chu Shang Show Chwan Hospital, Nantou, Taiwan; 2 Graduate Institute of Clinical Medicine, College of Medicine, National Taiwan University, Taipei, Taiwan; 3 Department of Urology, National Taiwan University Hospital and College of Medicine, National Taiwan University, Taipei, Taiwan; 4 Division of Nephrology, Department of Internal Medicine, National Taiwan University Hospital, Taipei, Taiwan; 5 Division of Cardiology, Department of Internal Medicine, National Taiwan University Hospital, Taipei, Taiwan

**Keywords:** Adrenalectomy, Hyperuricemia, Renal impairment, Primary aldosteronism

## Abstract

**Background:**

Primary aldosteronism (PA), which is present in 5–18% of hypertensive patients, is a leading cause of secondary hypertension. Adrenalectomy is often recommended for patients with unilateral primary aldosteronism (uPA), yielding good long-term outcomes. PA patients without hyperuricemia and chronic renal failure before adrenalectomy were enrolled in this cohort study. Serum uric acid (SUA) and renal filtration were measured one year post-adrenalectomy. Their relationships with pathologic features, histopathological subtype (classical or nonclassical (HISTALDO consensus)), and vessel stiffness were explored. The aim of this cohort study is to evaluate the correlation between post-adrenalectomy serum uric acid (SUA) levels and estimated glomerular filtration rate (eGFR) with the pathologic features delineated by the HISTALDO consensus. Additionally, the study seeks to assess the impact of these biochemical markers on peripheral vessel stiffness and brachial-ankle pulse wave velocity (baPWV) at a one-year follow-up visit.

**Methods:**

This prospective cohort study included patients (*N* = 100) diagnosed with uPA who underwent adrenalectomy from Jan 1, 2007 to Dec 31, 2022.

**Results:**

At follow-up, elevated SUA, hyperuricemia, and a > 25% eGFR decrease were significantly more common in the classical than the nonclassical group. The incidence of postoperative hyperuricemia, herein referred to as post-adrenalectomy hyperuricemia (PAHU), was 29% (29/100) overall, 34.8% (23/66) in the classical group and 17.6% (6/34) in the nonclassical group. The incidence of eGFR reduction > 25% was 33% (33/100), 43.9% (29/66), and 11.8% (4/34), respectively. baPWV decreased more in the classical group than the nonclassical group.

**Conclusion:**

For PA patients with PAHU and/or renal impairment, we suggest monitoring SUA, pH, urine uric acid, and urine crystals and performing a KUB study and peripheral vascular and renal sonography (on which pure uric acid stones in the KUB are radiolucent) to determine whether drug intervention is required for cases of asymptomatic PAHU, especially patients in male gender, classical histopathology, or renal impairment.

## Background

Primary aldosteronism (PA), first described by Dr. Jerome Conn in 1955, is a type of adrenal disorder characterized by abnormal autonomous aldosterone production. Since then, several subtypes of PA have been identified [1, 2].

PA is a leading cause of secondary hypertension and occurs in 5-18% of hypertensive patients [3]. In comparison with essential hypertension, the incidence and severity of cardiovascular and renal damage and metabolic disturbances is increased [4].

According to the histopathology of primary aldosteronism (HISTALDO) consensus, the histopathology of adrenal lesions in PA are further categorized into classical and nonclassical phenotypes via immunohistochemical examination of adrenal tumors by CYP11B2 to identify aldosterone-producing lesions [5, 6].

Adrenalectomy is often recommended for the treatment of unilateral primary aldosteronism (uPA) [11]. Meyer et al. reported that patients with classical uPA treated with adrenalectomy had better clinical outcomes postoperatively than those with nonclassical uPA [6].

Hyperuricemia and gout after adrenalectomy were first reported by Dr. Itskovitz in 1963 [7]. Here, we call this phenomenon postadrenalectomy hyperuricemia (PAHU), *pahu* meaning “drum” in Hawaiian. In our cohort study, we attempted to evaluate postadrenalectomy serum uric acid (SUA) and estimated glomerular filtration rate (eGFR) to correlate with the pathologic features based on the HISTALDO consensus and its impact on peripheral vessel stiffness and brachial-ankle pulse wave velocity (baPWV) at the one-year follow-up visit.

By examining these relationships, the study intends to provide insights into the biochemical and cardiovascular outcomes postadrenalectomy, potentially informing clinical practices and enhancing patient care.

## Methods

### Patient selections

We conducted a prospective cohort study that enrolled patients diagnosed with uPA who underwent adrenalectomy from Jan 1, 2007 to Dec 31, 2022. All patients received screening tests with or without confirmatory tests and, for unilateral PA. CT or MRI was used as a diagnostic test, with AVS serving as the standard of reference. To determine uPA using AVS, an Selective Index (SI) ≥ 2, which compares the adrenal vein cortisol level to the peripheral vein cortisol level, indicates successful adrenal vein sampling, ensuring accurate hormone measurements from the adrenal gland. Secondly, an Lateralization Index > 4 (LI), which compares the aldosterone to cortisol ratio between the dominant and non-dominant adrenal glands suggests significant asymmetry in aldosterone production, indicating unilateral disease. Therefore, unilateral PA is confirmed when the SI is ≥ 2 and the LI is > 4 between the dominant and non-dominant adrenal glands. “.

The data were accessed on March 1, 2023. All individuals with access to the information were unable to identify participants either during or after data collection. Written informed consent was obtained for the scientific study involving adrenal specimens, which included genotype analysis and histopathology. This study also involved the use of patient data, which was approved by the local ethics committee (approval number: 200611031R).

### Clinical measurements

Sex, age, Charlson comorbidity index, smoking status, body mass index (BMI), family history of hypertension, underlying diseases (cardiovascular event, diabetes and dyslipidemia), duration of hypertension, any episode of hypokalemia (< 3.6 mEq/L), and daily drug dose (DDD) of antihypertensive use were evaluated. In addition, perioperative measurements were obtained, such as plasma renin activity (PRA), plasma aldosterone concentration (PAC), systolic blood pressure (SBP), diastolic blood pressure (DBP), heart rate (HR), serum potassium (K), SUA, eGFR,, and baPWV. Patients with a history of gout, renal stones, chronic kidney disease (CKD) ≥ stage III (eGFR < 60) and hyperuricemia (male > 7, female > 6.5) were excluded.

PRA is typically assessed using the Gammacoat Plasma Renin Activity 125i Radioimmunoassay Ria Kit, supplied by DiaSorin Biotechnology. Samples are collected in the morning after a period of supine rest to standardize conditions, ensuring reliable results. PAC measurement utilizes the Aldosterone ELISA Kit by Cayman Chemical. Similarly, samples are collected in the morning following supine rest to minimize diurnal variations in aldosterone levels. Both tests necessitate strict adherence to timing protocols.

### Outcome assessment

One year after surgery, the outcome of uPA was assessed based on the primary aldosteronism surgery outcome (PASO) criteria. The criteria for biochemical success after surgery is to achieve a post-operative ARR of less than 10 (ng/dL per ng/mL/h) and a substantial percentage reduction from pre-operative ARR values around 50% or more is considered indicative of successful surgery. Successful outcomes are assessed not only by biochemical parameters but also by clinical improvements in blood pressure control and electrolyte balance.

The raw changes and the percentage changes in BMI, status of antihypertensive drug use, PRA, PAC, SBP, DBP, SUA, eGFR, and baPWV were compared between the two histopathological phenotype groups according to the HISTALDO consensus.

### Hyperuricemia

Hyperuricemia was defined as an SUA level greater than 7.0 mg/dL in men or greater than 6.5 mg/dL in women.

### Renal impairment and CKD

Postoperative renal impairment was defined as a postoperative eGFR decline of > 25% from the preoperative eGFR with repeat laboratory testing within 1 to 2 months at the one-year follow-up visit. CKD was defined as an eGFR < 60 mL/min/1.73 m2 on two different laboratory tests within 1 to 2 months of each other within the one-year follow-up.

### Histopathologic evaluation

All resected adrenal glands were paraffin-embedded and stained with HE and CYP11B2 IHC (cytochrome P450 aldosterone synthase). CYP11B2 immunostaining involves initial tissue preparation by fixing, embedding in paraffin, and sectioning onto slides. Antigen retrieval follows to expose CYP11B2 antigenic sites, with subsequent blocking to reduce nonspecific binding. Tissue sections are then incubated overnight at 4 °C with a primary antibody specific to CYP11B2. After primary antibody incubation, secondary antibodies conjugated with horseradish peroxidase-conjugated SignalStain Boost IHC Detection Reagent are applied. The detection is achieved using the 3,3-diaminobenzidine tetrahydrochloride Substrate Chromogen System, followed by hematoxylin counterstaining.

The determination of pathological classification follows the HISTALDO consensus. PA is classified into classical and non-classical types based on CYP11B2 immunostaining of the resected adrenal gland to identify the exact source of aldosterone overproduction. A dominant or solitary aldosterone-producing adenoma (APA) with strong CYP11B2 staining is classified as part of the classical group within the HISTALDO framework.

### Statistical analysis

Continuous variables with normal distribution were presented as mean (standard deviation [SD]); non-normal variables were reported as median (interquartile range [IQR]). Means of 2 continuous normally distributed variables were compared by independent samples Student’s t test. Mann-Whitney U test and Kruskal-Wallis test were used, respectively, to compare means of 2 and 3 or more groups of variables not normally distributed. The frequencies of categorical variables were compared using Pearson χ2 or Fisher’s exact test, when appropriate. The paired t test or the Wilcoxon signed rank test was used to compare values before and after adrenalectomy. We performed an ROC analysis to investigate the best cutoff values. Multivariate regression analyses were performed to identify risk factors *P* < 0.05 was considered statistically significant. All statistical analyses were conducted using IBM SPSS statistics 26 (IBM Corporation, Armonk, NY).

## Results

### Patient cohort and histopathology

This study consisted of 100 patients diagnosed with uPA comprising 40 male (40%) participants with a mean age of 54.6 ± 10.9 years and a mean duration of hypertension of 7.6 ± 7.4 years. Out of the 100 patients included in the study, 66 exhibited classical histopathology findings. Among the remaining 34 patients, nonclassical histopathology was observed, which included cases of MAPN with MAPM (18 patients), MAPM alone (13 patients), and MAPM with APDH (3 patients). Interestingly, among the 66 patients with classical histopathology, 46 were found to have MAPM or APDH in the adjacent cortex of either a solitary APA or a dominant APN.

The baseline characteristics of the patients stratified by histopathologic group are shown in Table 1. There were no significant differences in sex distribution, BMI, Charlson comorbidity index, current smoking status, family history of hypertension, underlying disease (DM, cardiovascular event, dyslipidemia), DDD in anti-hypertensives, or duration of hypertension between the classical and nonclassical groups. Furthermore, patients in the nonclassical histopathology group had significantly older age (60.1 ± 10.4 vs. 51.8 ± 10.1, *p* < 0.001), lower baseline PAC (39.6 ± 20.4 vs. 59.9 ± 35.1, *p* = 0.003), fewer episodes of hypokalemia (29.4% vs. 66.7%, *p* < 0.001) and less intense treatment with ACEIs/ARBs (20.6% vs. 62.1%, *p* < 0.001) than the classical group.

**Table 1:** Baseline characteristics of patients with primary aldosteronism according to histopathology subtype

Variable	ALL *N* = 100	Classical *N* = 66	Nonclassical *N* = 34	*p*-value
**Baseline Characteristics**				
**Age (years)**	54.6 ± 10.9	51.8 ± 10.1	60.1 ± 10.4	< 0.001
**Male**,** n (%)**	27 (39.7%)	17 (37.8%)	10 (43.5%)	0.697
**BMI (kg/m**^**2**^**)**	25.2 ± 3.8	25.3 ± 4.3	24.9 ± 2.8	0.625
**Charlson comorbidity index**	2.0 ± 1.6	2.2 ± 1.7	1.7 ± 1.5	0.151
**Current smoker**,** n (%)**	6(6%)	4 (6.7%)	2 (4.4%)	0.972
**Family history of hypertension**,** n (%)**	69 (69%)	49 (74.2%)	21 (65.6%)	0.197
**History of cardiovascular event**,** n (%)**	24 (24%)	15 (22.7%)	9 (26.5%)	0.678
**Diabetes**,** n (%)**	16 (16%)	12 (18.2%)	4 (11.8%)	0.407
**Dyslipidemia**,** n (%)**	24 (24%)	12 (18.2%)	12 (35.3%)	0.063
**Duration of hypertension (years)**	7.6 ± 7.4	7.4 ± 6.7	8.0 ± 8.7	0.703
**Medication use at confirmation period**				
**No. of antihypertensive drugs (DDD)**	2.0 ± 1.2	2.1 ± 1.2	1.7 ± 1.0	0.098
**α-blocker**,** n (%)**	25 (25%)	18 (27.3%)	7 (20.6%)	0.465
**β-blocker**,** n (%)**	45 (45%)	30 (45.5%)	15 (44.1%)	0.899
**ACEI /ARB**,** n (%)**	48 (48%)	41 (62.1%)	7 (20.6%)	< 0.001
**CCB**,** n (%)**	70 (70%)	45 (68.2%)	25 (73.5%)	0.580
**Diuretics**,** n (%)**	12 (12%)	6 (9.1%)	6(17.6%)	0.212
**Hypokalemia (< 3.6 mEq/L)**,** n (%)**	54(54%)	44 (66.7%)	10 (29.4%)	< 0.001
**Laboratory data**				
**PAC (ng/dL)**	53.1 ± 32.2	59.9 ± 35.1	39.6 ± 20.4	0.003
**PRA (ng/mL/hr)**	0.5 ± 0.8	0.6 ± 0.9	0.4 ± 0.6	0.246
**Serum potassium(mmol/L)**	3.7 ± 0.6	3.6 ± 0.6	3.9 ± 0.4	0.01
**eGFR (ml/min/1.73m**^**2**^**)**	106.2 ± 28.3	108.3 ± 31.4	101.9 ± 20.7	0.286
**SUA (mg/dl)**	5.1 ± 1.1	5.1 ± 1.0	5.2 ± 1.3	0.671
**SBP (mmHg)**	152.6 ± 17.4	151.9 ± 18.1	154.0 ± 16.0	0.569
**DBP (mmHg)**	91.0 ± 11.1	92.0 ± 11.0	89.5 ± 11.3	0.289
**baPWV(cm/sec)**	1667.6 ± 316.0	1636.2 ± 304.0	1730.3 ± 337.1	0.161
Clinical outcome*				0.046
**Complete success**	55 (55%)	41 (62.1%)	14 (41.1%)	
**Partial or Absent success**	45 (45%)	25 (37.9%)	20 (58.8%)	
**Biochemical outcome**				0.434
**Complete success**	91 (91%)	59 (89.4%)	32 (94.1%)	
**Partial or Absent success**	9 (9%)	7 (10.6%)	2 (5.88%)	

Data are presented as the mean and confidence interval for normally distributed data and the median and interquartile range for nonnormally distributed data

*PASO: Primary Aldosteronism Surgical Outcome

Abbreviations: baPWV: brachial-ankle pulse wave velocity, DBP: diastolic blood pressure; eGFR: estimated glomerular filtration rate; HR: heart rate; SBP: systolic blood pressure. PAC: plasma aldosterone concentration; PRA: plasma renin activity; SUA: serum uric acid

Note: To convert potassium from mmol/L to mEq/L, multiply by 1; to convert eGFR from mL/min to mL/s, multiply by 0.01667; to convert PAC from ng/dL to nmol/L, multiply by 0.02774; to convert PRA from ng/mL/hr to ng/(Lxs), multiply by 0.2778.

¶ Patients were withdrawn from antihypertensive medications at least 21 days before the study, except calcium antagonists or alpha-blockers.

### Postadrenalectomy renal function deterioration

**Fig. 1:**
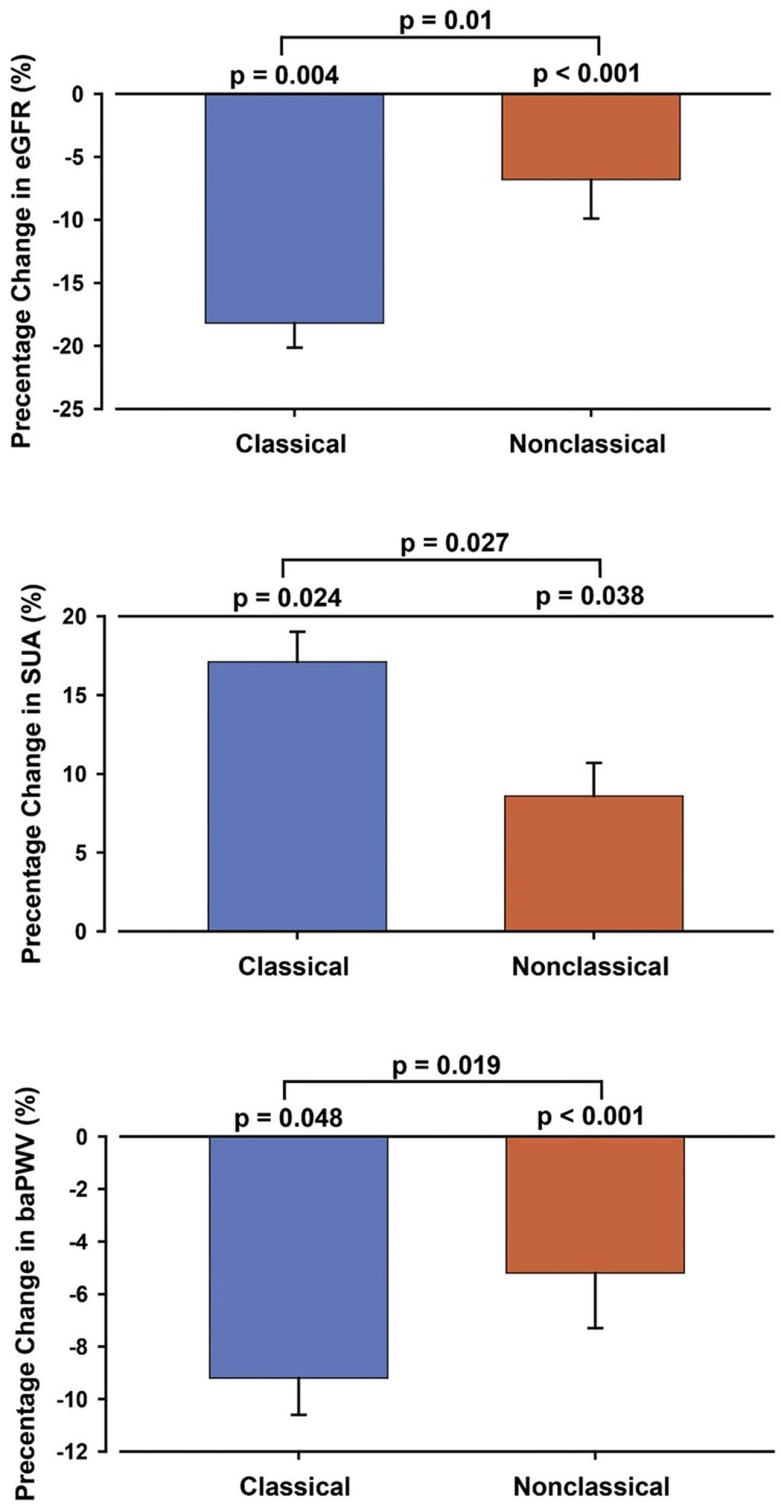
Postsurgical percentage change in eGFR (Fig. 1a), SUA (Fig. 1b), and baPWV (Fig. 1c) in patients with primary aldosteronism according to histopathology subtype

Table 1 summarizes the characteristics of patients categorized into classical and nonclassical groups before adrenalectomy. One year after adrenalectomy, the percentage change in eGFR was significantly reduced in the classical (-0.18 ± 0.16, *p* = 0.031) and nonclassical groups (-0.07 ± 0.18, *p* < 0.001) (Table 2), and the change also differed significantly between these two groups (Fig. 1a, *p* = 0.011).

**Table 2:** Characteristics at baseline and the 1-year post-adrenalectomy follow-up and the changes in magnitude and ratio of patients with primary aldosteronism according to histopathology subtype

Variable	Baseline	OP12M	*Δ	*P* value		**Δ in Ratio	*P* value
**Classical (** ***N*** ** = 66)**							
**SUA (mg/dl)**	5.1 ± 1.0	6.0 ± 1.5	0.9 ± 1.0	0.032		17.1 ± 15.6	0.024
**Serum potassium (mmol/L)**	3.6 ± 0.6	4.2 ± 0.4	0.6 ± 0.8	0.001		21.3 ± 28.5	0.002
**Glucose (mg/dL)**	99.0 ± 17.5	101.5 ± 20.3	2.5 ± 18.6	0.520		3.6 ± 20.5	0.498
**eGFR (ml/min/1.73m2)**	108.3 ± 31.4	86.6 ± 23.2	-21.8 ± 19.6	0.017		-18.2 ± 15.8	0.031
**PAC (ng/dL)**	59.9 ± 35.1	36.8 ± 26.3	-23.1 ± 40.2	0.039		-22.2 ± 59.1	0.938
**PRA (ng/mL/hr)**	0.6 ± 0.9	3.6 ± 2.4	3.1 ± 2.4	0.005		3503.2 ± 9108.6	0.005
**SBP (mmHg)**	151.9 ± 18.1	128.3 ± 16.8	-23.6 ± 18.3	< 0.001		-14.9 ± 11.1	< 0.001
**DBP (mmHg)**	92.0 ± 11.0	80.4 ± 10.7	-11.6 ± 11.3	< 0.001		-12.0 ± 11.7	< 0.001
**HR (bpm)**	72.2 ± 9.2	73.2 ± 12.4	1.0 ± 12.1	0.089		2.2 ± 17.0	0.293
**baPWV (cm/sec)**	1635.2 ± 283.9	1474.2 ± 278.0	-161.0 ± 205.0	0.026		-9.2 ± 11.5	0.035
**No. of antihypertensive drugs(DDD)**	2.1 ± 1.2	0.6 ± 0.8	1.5 ± 0.8	0.010			
**Nonclassical (** ***N*** ** = 34)**							
**SUA (mg/dl)**	5.2 ± 1.3	5.6 ± 1.5	0.4 ± 0.8	< 0.001		8.6 ± 12.2	< 0.001
**Serum potassium (mmol/L)**	3.9 ± 0.4	4.2 ± 0.3	0.3 ± 0.5	< 0.001		9.4 ± 13.5	< 0.001
**Glucose (mg/dL)**	106.0 ± 25.7	107.0 ± 33.8	2.0 ± 22.6	0.323		2.2 ± 20.2	0.216
**eGFR (ml/min/1.73m2)**	101.9 ± 20.7	94.5 ± 22.7	-7.4 ± 16.5	< 0.0001		-6.8 ± 18.0	< 0.001
**PAC (ng/dL)**	39.6 ± 20.4	28.5 ± 14.8	-11.5 ± 24.5	< 0.001		2.4 ± 140.2	0.015
**PRA (ng/mL/hr)**	0.4 ± 0.6	2.0 ± 2.5	1.6 ± 2.5	< 0.0001		2426.5 ± 3616.3	0.013
**SBP (mmHg)**	154.0 ± 16.0	137.6 ± 15.4	-16.5 ± 15.3	< 0.001		-10.3 ± 9.3	< 0.001
**DBP (mmHg)**	89.5 ± 11.3	81.0 ± 12.7	-8.5 ± 9.1	< 0.001		-9.3 ± 9.8	< 0.001
**HR (bpm)**	74.0 ± 14.1	69.8 ± 9.3	-4.3 ± 14.1	0.531		-3.4 ± 18.0	0.349
**baPWV (cm/sec)**	1746.0 ± 323.9	1632.8 ± 319.5	-101.0 ± 233.1	< 0.001		-5.2 ± 12.4	< 0.001
**No. of antihypertensive drugs(DDD)**	1.7 ± 1.0	0.6 ± 0.8	1.1 ± 0.8	0.010			

Data are presented as the mean and confidence interval for normally distributed data and the median and interquartile range for nonnormally distributed data

Abbreviations: baPWV: brachial-ankle pulse wave velocity, DBP: diastolic blood pressure; eGFR: estimated glomerular filtration rate; HR: heart rate; SBP: systolic blood pressure. PAC: plasma aldosterone concentration; PRA: plasma renin activity; SUA: serum uric acid

*Δ = post-pre

**Δ in ratio = (post-pre)/pre*100

Note: To convert potassium from mmol/L to mEq/L, multiply by 1; to convert eGFR from mL/min to mL/s, multiply by 0.01667; to convert PAC from ng/dL to nmol/L, multiply by 0.02774; to convert PRA from ng/mL/hr to ng/(Lxs), multiply by 0.2778.

¶ Patients were withdrawn from antihypertensive medications at least 21 days before the study, except calcium antagonists or alpha-blockers.

**Fig. 2:**
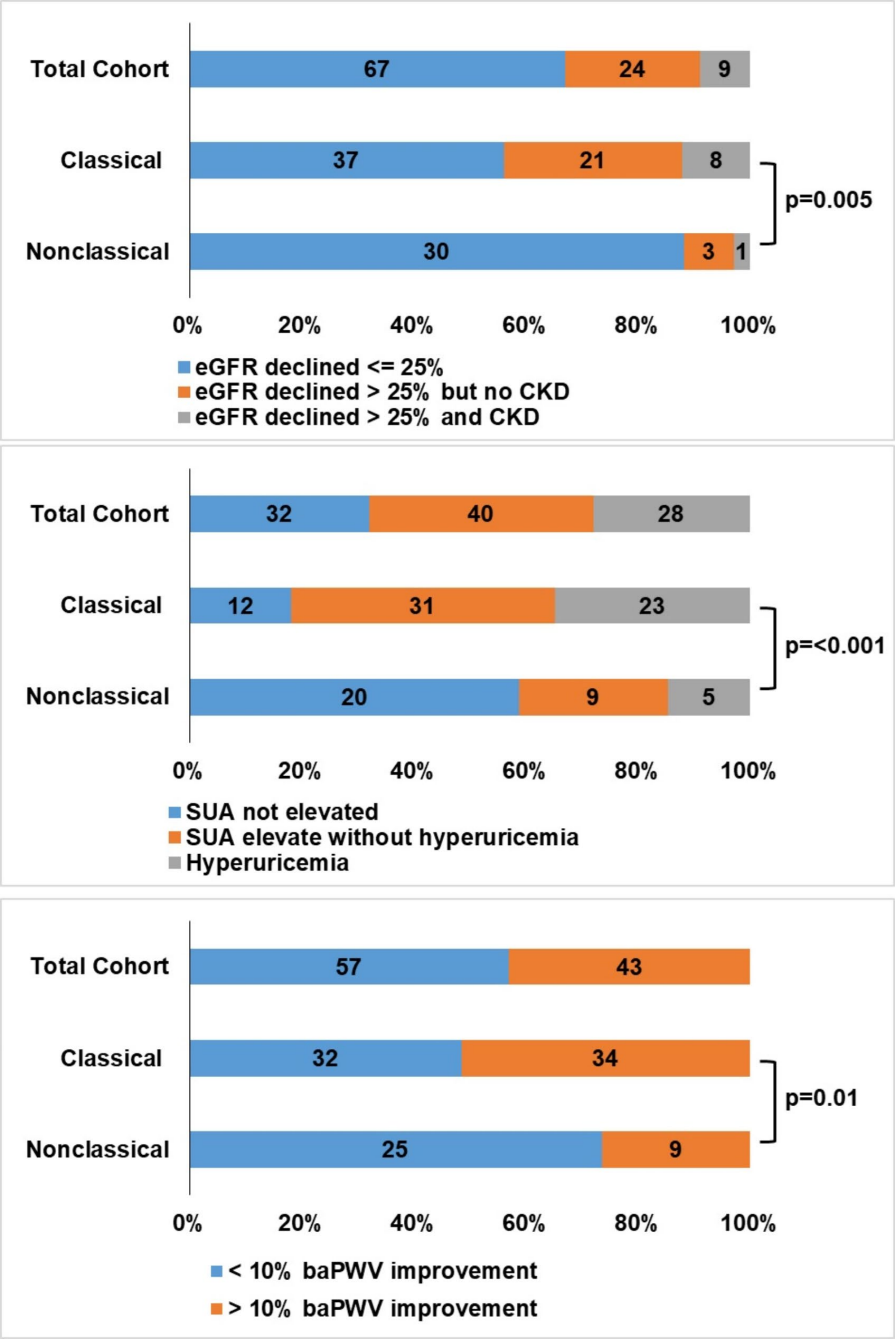
Postsurgical outcomes in terms of renal impairment (Fig. 2a), elevation of SUA (Fig. 2b), and > 10% reduction of baPWV (Fig. 2c) according to histopathology subtype

At the one-year follow-up, 29 patients (43.9%) in the classical group developed renal impairment. Of these, 8 patients had an eGFR < 60 mL/min/1.73 m2. Compared to those in the nonclassical group, only 1 out of 4 patients with renal impairment had an eGFR < 60 mL/min/1.73 m2 (Fig. 2a, *p* = 0.005).

We performed multivariate logistic regression controlled to explore the potential predictors of postoperative renal impairment; preoperative SBP (OR 1.06, 95% CI 1.04–1.08, *p* = 0.028) and type of adrenal histopathology (OR 5.59, 95% CI 2.39–13.08; *P* = 0.032) were risk factors for the development of renal impairment by 1 year after adrenalectomy (Table 3).

**Table 3:** Multivariate regression analysis of renal impairment, hyperuricemia, and > 10% reduction in baPWV in patients with PA at the one-year follow-up after adrenalectomy. Data are given as *P -*values for multivariable analysis

Variable	Odd Ratio(OR)	OR 95% CI	*p*-value
**Renal impairment**			
**Age (year)**	1.13	0.65–1.19	0.531
**Serum potassium**	0.72	0.44–1.18	0.429
**Plasma aldosterone concentration**	1.00	0.99–1.01	0.755
**SBP(mmHg)**	1.06	1.04–1.08	0.028
**Subtype of histopathology (nonclassical)**	5.59	2.39–13.08	0.032
**Hyperuricemia**			
**Age (year)**	1.04	1.00-1.08	0.206
**Serum potassium**	1.49	0.76–2.92	0.433
**Plasma aldosterone concentration**	1.01	0.99–1.02	0.425
**Gender (female)**	3.24	1.51–6.96	0.001
**Subtype of histopathology (nonclassical)**	3.70	1.61–8.46	0.032
**> 10% reduction of baPWV**			
**Age (year)**	0.98	0.95–1.01	0.372
**Serum potassium**	0.55	0.32–0.95	0.219
**Plasma aldosterone concentration**	1.05	1.03–1.08	0.028
**BMI**	0.89	0.83–0.96	0.108
**Subtype of histopathology (nonclassical)**	3.40	1.75–6.62	0.019

### Postadrenalectomy elevated serum uric acid and PAHU

One year after adrenalectomy, the percentage change in SUA was significantly positive in both the classical (0.17 ± 0.15, *p* = 0.024) and nonclassical groups (0.09 ± 0.12, *p* < 0.001) (Table 2), and the percentage change also significantly differed between groups (Fig. 1b, *p* = 0.038).

Overall, SUA was elevated in 68 (68%) of 100 patients at the one-year follow-up visit postadrenalectomy, and 28 (28%) patients had hyperuricemia (Fig. 2b). Fifty-four patients (81.8%) had elevated SUA, and 23 patients (34.8%) developed hyperuricemia in the classical group. In the nonclassical group, 14 out of 34 patients (44.1%) had elevated SUA, and 5 patients (14.7%) had hyperuricemia (Fig. 2b, *p* = 0.006).

Multivariate logistic regression showed the classical type of histopathology (OR 3.70, 95% CI 1.61–8.46; *P* = 0.032) and male sex (OR 3.24, 95% CI 1.51–6.96; *P* = 0.001) were risk factors for the development of PAHU by the 1-year visit (Table 3).

### Change in peripheral arterial stiffness after adrenalectomy

baPWV was elevated in both groups at baseline (Table 1). One year after adrenalectomy, the percentage change in baPWV was significantly reduced in the classical (-0.092 ± 0.115, *P* = 0.035) and nonclassical (-0.052 ± 0.124, p = < 0.001) groups (Table 2), and the percentage change differed between these two groups (Fig. 1c, *p* = 0.028).

Thirty-four patients (51.5%) in the classical group had a more than 10% reduction in baPWV, while only 9 patients (26.5%) achieved this outcome in the nonclassical group (Fig. 2c, *p* = 0.017).

After adjustments for age, serum potassium, and BMI, the multivariate logistic regression analysis showed that subtype of adrenal histopathology (OR 3.40, 95% CI 1.75–6.62; *P* = 0.019) and preoperative PAC (OR 1.05, 95% CI 1.03–1.08; *P* = 0.028) were correlated with a 10% baPWV reduction at 1 year after adrenalectomy (Table 3).

## Discussion

The study examines the less-explored areas of postoperative outcomes following adrenalectomy in primary aldosteronism (PA), focusing on hyperuricemia, renal impairment, and arterial stiffness one year post-surgery. These outcomes are evaluated based on the histopathological subtypes of adrenal tumors, distinguishing between classical and nonclassical types.

### Postadrenalectomy renal impairment in different subtypes of adrenal histopathology

Decreases in eGFR in PA patients after unilateral adrenalectomy are intriguing to clinicians. Postadrenalectomy renal insufficiency has been linked to preoperative renal hyperfiltration [8].

Our study found that histopathology subtype is a significant risk factor for developing renal impairment. At the one-year follow-up, 43.9% of patients in the classical group developed renal impairment compared to 11.8% in the nonclassical group. Multivariate logistic regression analysis confirmed that classical adrenal histopathology is an independent risk factor for postoperative renal impairment.

### Postadrenalectomy hyperuricemia in different subtypes of adrenal histopathology

PAHU, historically of limited interest, showed that hyperuricemia could result from severe hypertension and reduced uric acid (UA) excretion due to renal tubular alterations [7].

A previous study found lower UA levels in PA patients associated with higher PAC and lower plasma renin activity [9]. The classical group may experience a higher degree of UA rebound due to decreased aldosterone after uPA. Ogata et al. showed that excess aldosterone in PA causes endothelial damage, vascular stiffness, and glomerular damage, leading to increased glomerulosclerosis and postoperative eGFR decline [10].

Our study observed increased SUA and decreased eGFR at the one-year follow-up after adrenalectomy, suggesting that reduced UA excretion contributes to postadrenalectomy hyperuricemia. The classical group exhibited a higher incidence of hyperuricemia, likely due to severe hypertension and renal impairment.

### Postadrenalectomy arterial stiffness changes in different subtypes of adrenal histopathology

Patients with hyperaldosteronism experience increased arterial stiffness due to excess aldosterone and secondary hypertension [11].

Our study found that 51.5% of classical group patients had a > 10% reduction in baPWV one year post-surgery, compared to 26.5% in the nonclassical group. Multivariate regression revealed that classical adrenal histopathology and higher preoperative PAC are independent predictors of significant baPWV reduction post-adrenalectomy.

### Hyperuricemia and renal impairment

The causal relationship between hyperuricemia and kidney disease progression remains debated. Autopsy reports on gouty nephropathy patients often reveal impaired renal function, showing condiions like arteriolar sclerosis and urate crystal deposition. Observational studies generally indicate that elevated serum uric acid (SUA) independently predicts renal impairment and that reducing SUA levels can slow this progression [12–14]. Experimental models support that high UA levels cause glomerular injury and renal hypertension [15–17].

Hyperuricemia is prevalent in 60% of CKD patients, with gout attacks occurring in 24% [18, 19]. Medical intervention for asymptomatic hyperuricemia in CKD may be justified due to risks like nephrolithiasis and elevated intracellular UA levels [20–23]. The PERL study found that allopurinol did not significantly slow CKD progression in type 1 diabetes patients, contrasting with earlier studies suggesting benefits of lowering UA levels [24]. Differences may stem from PERL’s specific focus and rigorous design versus broader CKD patient inclusion in other studies.

**Fig. 3:**
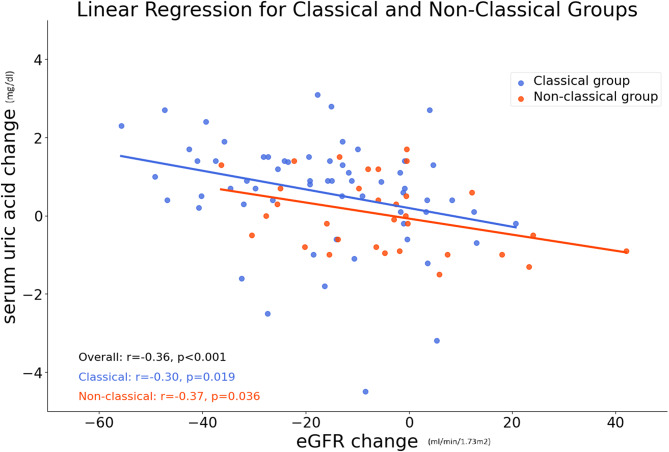
Linear regression for the change of serum uric acid and eGFR for classical and non-classical groups one year after target adrenalectomy

Our study focuses on post-adrenalectomy declines in eGFR leading to elevated UA, differing from PERL’s emphasis on UA reduction. Of 100 patients, 33 developed postoperative renal impairment, with 9 progressing to CKD. Thirteen of these had hyperuricemia, including six CKD patients. Patients with classical PA histology exhibited more severe disease, contributing to a greater decline in eGFR and higher UA levels post-adrenalectomy, likely due to the severity of their condition. Figure 3 illustrates the correlation between elevated SUA and eGFR decline for classical and non-classical groups.

### Elevated SUA and vessel stiffness

In 1992, Namba et al. studied UA metabolism in PA patients (16 patients, 8 post-adrenalectomy) compared to normotensive or essential hypertension (EHT) participants [25]. They found SUA levels increased significantly after adenoma removal but remained lower than in the NT or EHT groups, likely due to the short follow-up period (2 weeks). Many studies show aldosterone and UA affect vascular elasticity. In our study, aldosterone levels dropped rapidly after adenoma removal, and baPWV improved more in the classical group than the nonclassical group one year post-surgery. However, the rise in UA levels was not dramatic, possibly due to the lack of blood vessel elasticity deterioration at the one-year follow-up.

Follow-up duration is crucial for observing hyperuricemia trends. Namba et al. found SUA elevated at 2 weeks post-adrenalectomy but lower than in NT and EHT patients. Itskovitz’s 7–8 year follow-up diagnosed two-thirds of patients with hyperuricemia and 10.5% with gout attacks. Our one-year follow-up showed 69% had elevated SUA levels, 29% had hyperuricemia, with a high proportion in the classical group. The greater reduction in baPWV in the classical group suggests that elevated UA’s impact on vessel stiffness may require a longer follow-up period to be fully understood.

### Clinical significance

Most studies focus on decreased SBP and antihypertensive medication to evaluate patient outcomes. Our findings recommend that clinicians closely monitor SUA, pH, urine uric acid, and urine crystals. Additionally, performing KUB studies and peripheral vascular and renal sonography is crucial to determine if drug intervention is needed for asymptomatic PAHU, especially in male patients, those with classical histopathology, or renal impairment.

Our study highlights the importance of managing uPA in patients. Adrenalectomy, aimed at removing excess aldosterone, is suitable for both classical and nonclassical subtypes. Postoperative improvements in vessel stiffness support this approach. However, the higher incidence of postadrenalectomy renal insufficiency and hyperuricemia in the classical group requires clinicians to pay extra attention to SUA and the risk of gout in these patients.

Hyperuricemia following adrenalectomy can be attributed to multiple interrelated mechanisms, particularly those associated with hormonal and renal changes that occur after the removal of the adrenal glands. A crucial mechanism involves the renin-angiotensin-aldosterone system. Adrenalectomy can disrupt this system by reducing aldosterone production, which plays a critical role in maintaining potassium and sodium balance. Pre-adrenalectomy, aldosterone-induced glomerular hyperfiltration can mask underlying renal dysfunction in patients with PA. Following adrenalectomy, the decrease in aldosterone leads to a redistribution of blood flow, unmasking previously hidden renal dysfunction. This shift can exacerbate difficulties in uric acid excretion, contributing to the development of hyperuricemia post-surgery. Additionally, the hormonal imbalance triggered by adrenalectomy often leads to compensatory mechanisms, such as an increase in adrenocorticotropic hormone from the pituitary gland. This increase in adrenocorticotropic hormone can indirectly influence other hormones and enzymes involved in purine metabolism, potentially increasing uric acid production.

The severity of pre-existing conditions, such as hypertension and aldosterone excess, also significantly impacts the development of hyperuricemia after adrenalectomy. Severe hypertension, commonly seen in patients with primary aldosteronism, can lead to chronic kidney damage over time. Chronic kidney disease is a well-established risk factor for hyperuricemia, as impaired kidney function reduces the excretion of uric acid. Even though blood pressure typically decreases significantly after adrenalectomy, the pre-existing renal impairment from long-standing severe hypertension may still hinder the kidneys’ ability to excrete uric acid efficiently, thereby increasing the risk of hyperuricemia.

Similarly, the degree of aldosterone excess before surgery plays a crucial role. High levels of aldosterone can cause increased potassium excretion and sodium retention, leading to hypokalemia. Hypokalemia, in turn, can impair renal excretion of uric acid. The more severe the aldosterone excess before surgery, particularly in classical-type cases, the greater the likelihood of significant hypokalemia and consequent hyperuricemia. Following adrenalectomy, the abrupt drop in aldosterone levels can lead to rapid shifts in electrolyte balance, potentially worsening hyperuricemia, particularly in patients with severe aldosterone excess.

A key factor is the significant decrease in cortisol production, especially in the 4–16% of classical-type cases with primary hyperaldosteronism that co-secrete cortisol [26]. Cortisol typically inhibits the reabsorption of uric acid in the kidneys; therefore, a reduction in cortisol levels post-surgery can lead to increased reabsorption of uric acid, causing hyperuricemia. Moreover, the adrenal glands are responsible for producing catecholamines like epinephrine and norepinephrine. After adrenalectomy, the reduction in these hormones can decrease renal blood flow and glomerular filtration rate, impairing the clearance of uric acid and leading to its accumulation in the blood. Uric acid, often linked to the negative effects of hyperuricemia, also acts as an antioxidant by scavenging reactive oxygen species like singlet oxygen [27]. This dual role suggests that while elevated uric acid levels post-adrenalectomy are risky, they might offer some protection against oxidative stress. Recent studies highlight the complex effects of treatments for PA on kidney function. Wu et al. [28] found that PA patients had higher kidney damage markers at diagnosis, and while kidney tubule health improved post-treatment, a decline in estimated glomerular filtration rate likely reflected hemodynamic changes. Sheu et al. [29] observed that an estimated glomerular filtration rate dip post-therapy is linked to increased mortality and cardiovascular events, emphasizing the eed for close renal monitoring after adrenalectomy to manage hyperuricemia’s dual effects.

Strengths of our study include reliable histopathology classification with CYP11B2 IHC staining and a high one-year follow-up rate. Limitations include the exclusion of patients with missing data, lack of long-term follow-up data, and incomplete urinary parameters for metabolic analysis.

## Conclusions

This study underscores the importance of adrenal histopathology subtypes in predicting postoperative outcomes such as renal impairment, hyperuricemia, and arterial stiffness. Targeted unilateral adrenalectomy for uPA shows promising improvements in vessel stiffness. However, hyperuricemia after adrenalectomy is influenced by a complex interplay of hormonal changes, renal function, and the severity of pre-existing conditions such as hypertension and aldosterone excess. Patients with more severe forms of these conditions are at a higher risk of developing hyperuricemia post-surgery. Therefore, careful monitoring of renal function, electrolyte balance, and uric acid levels is essential after adrenalectomy to manage and mitigate the risk of hyperuricemia effectively. Proper post-operative care, including hormone replacement therapy and renal function support, may be necessary to prevent or manage these complications. Future research should focus on longer-term follow-up to understand better the progressive effects of hyperuricemia and aldosterone-related metabolic disturbances on renal and cardiovascular health.

## Data Availability

This study used the data under the authorization of Taiwan Primary Aldosteronism Investigation (TAIPAI) Study Group, however, the interpretations and conclusions contained herein do not represent the opinions of the aforementioned group. In addition, this group had no role in study design, data collection and analysis, decision to publish, or preparation of the manuscript. Vin-Cent Wu (email: dr.vincentwu@gmail.com; ntu007abao@gmail.com) should be contacted if anyone wants to request the data from this study.

## Abbreviations

AF: Atrial fibrillation
APA: Aldosterone-producing adenoma
baPWV: Brachial artery pulse wave velocity
BMI: Body mass index
CKD: chronic kidney disease
DBP: diastolic blood pressure
DDD: daily drug dose
EHT: essential hypertension
HISTALDO: histopathology of primary aldosteronism
HR: heart rate
IHC: immunohistochemistry
PAHU: postadrenalectomy hyperuricemia
PA: primary aldosteronism
PASO: primary aldosteronism surgery outcome
PAC: plasma aldosterone concentrations
PRA: Plasma renin activity
SUA: Serum uric acid
uPA: Unilateral primary aldosteronism
